# Supplementary material for: Effects of Dietary Anaplerotic and Ketogenic Energy Sources on Renal Fatty Acid Oxidation Induced by Clofibrate in Suckling Neonatal Pigs
Source: Int J Mol Sci. 2020 Jan 22;21(3):726. doi: 10.3390/ijms21030726 (PMC7037708; doi:10.3390/ijms21030726)
Supplement: Supplementary file 1 [file ijms-21-00726-s001.pdf]

Supplementary Materials

Effects of Dietary Anaplerotic and Ketogenic Energy Sources on Renal Fatty Acid Oxidation Induced by Clofibrate in Suckling Neonatal Pigs

Xi Lin [1,*], Brandon Pike [1], Jinan Zhao [1,†], Yu Fan [1,‡], Yongwen Zhu [1,§], Yong Zhang [1,‖], Feng Wang [1] and Jack Odle [1]

[1] Laboratory of Developmental Nutrition, Department of Animal Sciences, North Carolina State University, Raleigh, NC 27695, USA; bepike2@ncsu.edu (B.P.); jnzhao@zinpro.com (J.Z.); fanyucau@163.com (Y.F.); zhuyw@scau.edu.cn (Y.Z.); yongzhang208@163.com (Y.Z.); fwang22@ncsu.edu (F.W.); jodle@ncsu.edu (J.O.)

[*] Correspondence: xilin@ncsu.edu; Tel.: +01-919-515-4014

[†] Present Address: Sales Department, Zinpro Corporation, Eden Prairie, MN 55344, USA

[‡] Present Address: College of Animal Science and Technology, China Agricultural University, Beijing 100094, China

[§] Present Address: Guangdong Provincial Key Laboratory of Animal Nutrition and Regulation, College of Animal Science, South China Agricultural University, Guangzhou 510000, China

[‖] Present Address: College of Biological Engineering, Henan University of Technology, Zhengzhou 450001, China

Table S1 Relative gene expression

	Clofibrate					Medium-chain fatty acid					
FA	N	Clo+	Clo-	SEM	*p*-V	Tri2MPA	GlySUC	TriC5	TriC6	SEM	*p*-V
						Fold					
PPARα	1.00	1.43	1.07	0.24	0.27	1.03	1.44	1.43	1.11	0.33	0.31
MCD	1.00	1.28	1.00	0.23	0.38	1.13	1.29	1.18	0.97	0.33	0.38
KCoA	1.00	1.15	1.10	0.11	0.76	1.24	1.04	1.14	1.10	0.16	0.76
PGC1	1.00	1.61	1.35	0.30	0.56	1.21	2.06	1.67	0.98	0.43	0.56
VLCAD	1.00	1.32	1.03	0.15	0.21	1.11	1.45	1.32	0.82	0.22	0.21
FGF21	1.00	1.17	1.47	0.22	0.35	1.54	1.37	1.29	1.09	0.32	0.35
MCAD	1.00	4.85	3.84	1.99	0.71	3.46	1.98	5.43	6.51	2.70	0.71
LCAD	1.00	1.14	1.20	0.14	0.77	2.48	3.16	2.82	2.07	0.332	0.48

Data are least square means with standard errors (n=5-7). MCD: Malonyl-CoA decarboxylase; KCoA: 3-kotoaceyl-CoA thiolase; PGC1α: PPARG Coactivator alpha; VLCAD: Very long-chain acyl-CoA dehydrogenase; FGF21: Fibroblast growth factor 21; MCAD: Medium-chain acyl-CoA dehydrogenase; LCAD: Long-chain acyl-CoA dehydrogenase; PPARα: Peroxisome proliferator-activated receptor alpha.

Table S2 Primers

Genes	Forward primer(5'-3'), Sen	Reverse primer(5'-3'), Anti	Amplicon Size, bp	NCBI (Gene Bank)

MCD	TCT GAG GCT GTG CAT CCC GTT AAA	AGA GAA GAA GTA GCA CCT GCG GTA	81	AK235264
KCoA	CAG TTT GAT GTG GTT GTG GC	GCA GGG AGC TCA GGT GAT AG	173	AF028007
PGC1α	GCA GTT CTC ACA GAG ACG CT	TAG AGA CGG CTC TTC TGC CT	162	NM_213963.2; Sus
VLCAD	AGA GCG TTG ACG TTC CC	GCT GGC AGG CAT TTG AC	105	AF022255.1
FGF21	ACT GTG GGT CCC TGT GCT G	ATC CGT GTA GAG GTA TCG TTG G	118	NM_001163410.1 sus
MCAD	GGC CAA CGA TGT TCA GAT ACA A	GGT ATT TCG GCG ACC AGA ATC	333	NM_214039.1; sus
LCAD	TTG GAG GGG ACT TGT ACT CG	CCA TCC TTC TTG GCA TTT GT	247	NM_213897.1; sus
PPARα	GCC CAA GTT TGA CTT CGC CAT GAA	ATG CAC GAT ACC CTC CTG CAT TCT	151	DQ437887

MCD: Malonyl-CoA decarboxylase; KCoA: 3-kotoacyl-CoA thiolase; PGC1α: PPARG Coactivator alpha; VLCAD: Very long-chain acyl-CoA dehydrogenase; FGF21: Fibroblast growth factor 21; MCAD: Medium-chain acyl-CoA dehydrogenase; LCAD: Long-chain acyl-CoA dehydrogenase; PPARα: Peroxisome proliferator-activated receptor alpha.
